# Special Issue “Efficacy and Safety of Antiviral Therapy”

**DOI:** 10.3390/v15071411

**Published:** 2023-06-21

**Authors:** Agnese Colpani, Andrea De Vito, Giordano Madeddu

**Affiliations:** Unit of Infectious Diseases, Department of Medicine, Surgery, and Pharmacy, University of Sassari, 07100 Sassari, Italy

This comprehensive collection of papers contains a wide range of studies and observations centered on antiviral therapies, with a particular focus on HIV and other viral infections such as monkeypox and SARS-CoV-2. These studies shed light on various aspects of antiviral therapies, including treatment efficacy and tolerability. This Special Issue contains 13 original studies investigating different aspects of antiviral therapies; one is a preclinical study performed on mice, and the others are clinical studies.

The preclinical study conducted by Gunder et al. investigates the effectiveness of the topical protease inhibitor Saquinavir (SQV) in preventing anal cancer in mice with established anal dysplasia. Their findings highlight the potential of topical protease inhibitors as a preventive measure for anal cancer [1].

The clinical studies concern three different viruses: SARS-CoV-2, HIV, and MPox.

Regarding the prevention of COVID-19, we have learned more about the efficacy of early antiviral therapies. The studies presented by Del Borgo et al. and De Vito et al. [2,3] evaluated the efficacy of antiviral therapies, confirming the results from the available literature [4,5]. Rombini et al., on the contrary, evaluated the efficacy of a Nucleoside Reverse Transcriptase Inhibitor (NRTI) already approved for the treatment of HIV. In recent years, some in vitro and in vivo studies have hypothesized about the efficacy of HIV antiretroviral treatment in preventing the progression of COVID-19 [6,7,8].

Unfortunately, many people with SARS-CoV-2 infection developed severe COVID-19, and many of them died. Despite the prevalence of deaths due to COVID-19 constantly reducing, exploring existing treatments is crucial for future outbreaks. Marocco et al. reported data on the efficacy of remdesivir, an NRTI, in people with COVID-19 who required oxygen supplementation [9]. Mussini et al. reported data on the use of glucocorticoids plus tocilizumab (an IL-6 antagonist), which showed good efficacy under certain conditions [10].

Regarding MPox, this Special Issue presents the findings by Candela et al., who delve into the identification and characterization of monkeypox virus strains [11]. By analyzing the genetic makeup and evolutionary patterns of different monkeypox strains, this research contributes to a better understanding of the virus’s epidemiology and potential implications for public health.

Finally, six papers explored the efficacy and safety of different antiretroviral treatments for individuals living with HIV. These studies investigate the impact of various antiretroviral drugs on viral load suppression and immune recovery. Furthermore, they explore the effects of these treatments on lipid profiles, providing crucial insights into the potential metabolic implications for HIV patients. Hidalgo-Tenorio et al. and Lee et al. focused on the efficacy and safety of dual regimens—rilpivirine/dolutegravir and lamivudine/dolutegravir, respectively [12,13]. Hidalgo-Tenorio et al. found a very low percentage of virological failure and excellent durability. Lee et al. yielded an even better result, observing no virological failure nor therapeutic switch. These two studies confirm that dolutegravir-based dual regimens have excellent safety and efficacy in experienced people living with HIV in “real life” [14,15].

Fabbiani et al. focused on naïve people with low CD4 cells count, treated with a dolutegravir or boosted darunavir-containing regimens [16]. They found similar efficacies in AIDS- and late-presenting patients. A higher risk of treatment discontinuation due to central nervous system toxicity was observed with dolutegravir; conversely, they observed a higher probability of treatment simplification with darunavir.

Lazzaro et al. reported their data on the efficacy, safety, and tolerability of the bictegravir-containing regimen, specifically focusing on people older than 55 with a 2-year follow-up [17]. Theyconcluded that this regimen is effective, safe, and well-tolerated in PLWH.

Iannone, instead, reported data on the use of doravirine, the new-generation non-nucleoside reverse-transcriptase inhibitor, finding good efficacy and a favorable profile on lipid metabolism at 48 weeks of follow-up [18].

Lastly, De Vito et al. focus on treatment interruption in individuals who have been living with HIV for over 20 years. By analyzing the reasons behind treatment switches, this study uncovers factors influencing treatment adherence and interruption patterns [19]. Understanding these factors can inform healthcare providers and policymakers to design interventions to enhance treatment adherence and long-term health outcomes for HIV patients.

In conclusion, this collection of articles offers valuable insights for the evaluation of different treatment strategies, clinical outcomes, and associated factors in the context of infectious diseases. By examining various aspects of HIV, monkeypox, and COVID-19, these studies contribute to our understanding of disease management, antiviral therapies, and potential implications for public health. The findings presented in these papers may pave the way for improved treatment approaches, enhanced patient outcomes, and the better control of infectious diseases in the future. Finally, we would like to acknowledge all authors for their contributions to this Special Issue. It has been a pleasure to read and learn from their excellent work. Together, we will continue our research to discover and better understand the efficacy and safety of antiviral therapies.
